# Carvacrol instigates intrinsic and extrinsic apoptosis with abrogation of cell cycle progression in cervical cancer cells: Inhibition of Hedgehog/GLI signaling cascade

**DOI:** 10.3389/fchem.2022.1064191

**Published:** 2023-01-11

**Authors:** Afza Ahmad, Rohit Kumar Tiwari, Mohd Saeed, Hadba Al-Amrah, Ihn Han, Eun-Ha Choi, Dharmendra K. Yadav, Irfan Ahmad Ansari

**Affiliations:** ^1^ Department of Biosciences, Integral University, Lucknow, India; ^2^ Department of Biology, College of Sciences, University of Hail, Hail, Saudi Arabia; ^3^ Department of Biological Sciences, Faculty of Science, King Abdulaziz University, Jeddah, Saudi Arabia; ^4^ Plasma Bioscience Research Center, Applied Plasma Medicine Center, Department of Electrical & Biological Physics, Kwangwoon University, Seoul, South Korea; ^5^ Department of Pharmacy and Gachon Institute of Pharmaceutical Science, College of Pharmacy, Gachon University, Incheon, South Korea

**Keywords:** beta-glucan, antioxidant activity, apoptosis, anticancer, cervical cancer, ROS generation

## Abstract

Recent times have seen a strong surge in therapeutically targeting the hedgehog (HH)/GLI signaling pathway in cervical cancer. HH signaling pathway is reported to be a crucial modulator of carcinogenesis in cervical cancer and is also associated with recurrence and development of chemoresistance. Moreover, our previous reports have established that carvacrol (CAR) inhibited the proliferation of prostate cancer cells *via* inhibiting the Notch signaling pathway and thus, it was rational to explore its antiproliferative effects in cervical cancer cell lines. Herein, the present study aimed to investigate the anticancer and apoptotic potential of CAR on C33A cervical cancer cells and further explore the underlying mechanisms. We found that CAR significantly suppressed the growth of C33A cells, induced cell cycle arrest, and enhanced programmed cell death along with augmentation in the level of ROS, dissipated mitochondrial membrane potential, activation of caspase cascade, and eventually inhibited the HH signaling cascade. In addition, CAR treatment increased the expression of pro-apoptotic proteins (Bax, Bad, Fas-L, TRAIL, FADDR, cytochrome c) and concomitantly reduced the expression of anti-apoptotic proteins (Bcl-2 and Bcl-xL) in C33A cells. CAR mediates the activation of caspase-9 and -3 (intrinsic pathway) and caspase-8 (extrinsic pathway) accompanied by the cleavage of PARP in cervical cancer cells. Thus, CAR induced apoptosis by both the intrinsic and extrinsic apoptotic pathways. CAR efficiently inhibited the growth of cervical cancer cells *via* arresting the cell cycle at G0/G1 phase and modulated the gene expression of related proteins (p21, p27, cyclin D1 and CDK4)**.** Moreover, CAR inhibited the HH/GLI signaling pathway by down regulating the expression of SMO, PTCH and GLI1 proteins in cervical carcinoma cells. With evidence of the above results, our data revealed that CAR treatment suppressed the growth of HPV^−^C33A cervical cancer cells and further elucidated the mechanistic insights into the functioning of CAR.

## 1 Introduction

With approximately 600,000 newly diagnosed cases and 342,000 demises reported globally during 2020, cervical carcinoma remains the fourth most routinely diagnosed cancers in women and the fourth leading cause of mortality and morbidity in females (Sung et al., 2021). Persistent infection with Human papilloma Virus (HPV) (subtypes 16, 18, 31, 33, 35, 39, 45, 51, 52, 56, 58, and 59) along with other risk factors, including increased parity, infection with HIV and smoking causes cervical cancer (Bosch et al., 2002). Regional discrepancies in the burden of cervical cancer are bleak and reveal the availability, coverage, and quality of preventative interventions and the prevalence of risk factors. Approximately 9/10 women who dies from cervical cancer live in low- and middle-income countries (L/MICs). Inequities are broadening since high-income countries have witnessed a steep decline in the incidence rates, along with some nations moving ahead towards eliminating cervical carcinoma in the upcoming decades (Simms et al., 2019). In contrast, the incidence rate has increased in some sub-Saharan African countries. In these nations, the rate of incidence have either increased or remained mostly at high levels in various eastern European and west-Asian countries (Arbyn et al., 2011; Vaccarella et al., 2017; Arbyn et al., 2020).

Although the management of worldwide screening programs has reduced the occurrence and mortality of cervical carcinoma, however, the incidence of this dreaded disease within the young female population remains a grievous public health concern. Moreover, the systemic use of chemotherapeutic drugs leads to the development of drug resistance which eventually results in poor gynecological outcomes (Moss and Kaye, 2002; Lin et al., 2016). However, there is a constant need of novel drug development which could combat with drug resistance and adverse aftermath associated with the current treatment (Datta et al., 2019).

Phytocompounds are usually non-toxic, proven effective against numerous diseases, and considered a safe, cheap and effective alternative against cancer (Lai and Roy, 2004). Carvacrol (CAR) is a phenolic monoterpenoid abundantly present in the essential oils of oregano and thyme and is well-known for exerting multiple pharmacological effects such as antimicrobial, anticancer, insecticidal, anti-angiogenic, and anti-tumor activity (Sökmen et al., 2004; Baser, 2008). Notably, the Food and Drug Administration (FDA) has authorized the usage of CAR as a food supplement which testifies its non-toxic nature (Zotti et al., 2013). Furthermore, it is reported that CAR exerts cytotoxic effects on breast, lung, and colon cancer cells; however, the effect of CAR on the proliferation and apoptosis of cervical cancer and its underlying mechanism is not deciphered yet (Dai et al., 2016).

Infection with HPV is regarded as an initial strike that causes cervical carcinoma. Despite that, this factor is not individually sufficient for cancer development. Several additional cellular alterations are needed to commend the action of HPV. Correspondingly, in the present report, we have investigated the effect of CAR on the functionality of Hedgehog (HH) signaling in cervical cancer cells (Samarzija and Beard, 2012). The HH signaling cascade has been demonstrated to play an imperative role in the proliferation, metastasis, recurrence, invasion, drug resistance, and radioresistance of cervical cancer (Liu and Wang, 2019). The binding of HH ligand to its receptor patched (PTCH) activates the HH pathway. This binding relieves the repression from its second receptor Smoothened (SMO), which relocates to the cell membrane and drives a range of reactions leading to the translocation of transcription activators encoded by Glioma associated oncogenes (GLI1, GLI2 and GLI3) into the nucleus and subsequent transcription of target genes (Wu et al., 2020). Previous studies have established that molecular alterations in HH signaling cascade leads to various cancers such as medulloblastoma, basal cell carcinoma, small cell lung cancer, and prostate cancer. Reportedly, HH pathway is found to be hyper activated in cervical cancer and is associated with poor prognosis (Wang et al., 2010).

In this report, we studied the effect of CAR on regulating HH signaling cascade in HPV^−^ C33A cervical cancer cells *via* apoptosis induction and abrogation of cell cycle progression. However, to the best of our knowledge, we are reporting for the first time that CAR suppresses the progression of cervical cancer by inhibiting the HH signaling pathway.

## 2 Materials and methods

### 2.1 Reagents and chemicals

Carvacrol (CAR), DAPI (4, 6-diamidino-2-phenylindole), propidium iodide (PI), and 2, 7-dichlorodihydrofluorescein diacetate (DCFH-DA) were purchased from Sigma (St. Louis, MO, United States). Caspase-9, -8 and -3 colorimetric assay kit with catalogue numbers K119, K113–25 and K106-100 were procured from BioVision, United States. Acridine orange, Ethidium bromide, RPMI-1640, fetal bovine serum (FBS), 1% antibiotic-antimycotic solution, RNase A, 3-(4,5-dimethylthiazol-2-yl)-2,5-diphenyl tetrazolium bromide (MTT) and HiPurATM Total RNA Miniprep Purification Kit were purchased from Himedia India, Ltd., Mumbai, India. JC-1 mitochondrial membrane potential (MMP) assay kit was purchased from G-Biosciences, United States. All the primer sequences utilized during the study were procured from IDT, United States. FITC Annexin V Apoptosis Detection Kit was procured from BD Bioscience, PharMingen (San Diego, United States of America). DyNAmoColorFlash SYBR Green qPCR Kit and Verso cDNA synthesis kit were obtained from Thermo-Scientific, United States.

### 2.2 Cell line maintenance

Human cervical cancer cell line (C33A) was procured from the national repository division of the National Centre for Cell Sciences (NCCS), Pune, India. C33A cells were grown and maintained in RPMI-1640 completed with FBS (10%) and antibiotic-antimycotic solution (1%) under optimal culture conditions (temperature: 37 °C and 5% CO2).

### 2.3 Methods

#### 2.3.1 Cell proliferation assay

Cell viability was assessed by using MTT assay as described previously (Ansari et al., 2021). Briefly, C33A cells were cultured in 96-well plates (5 × 10^3^cells/well). C33A cells were maintained in the presence of CAR for 24 and 48 h. 10 µl MTT working dye (5 mg/ml) was added to the cultured C33A cells and then incubated for an additional 4 h. After that, media was removed and 100 μl DMSO was added to dissolve MTT crystals. Absorbance intensity was analyzed at 570 nm by an ELISA microplate reader (Bio-Rad, United States of America). Viability of C33A cells was calculated as a ratio of the optical density of treated and untreated cells.

#### 2.3.2 LDH assay

Human cervical cancer cells (C33A) were cultured into a 96-well plate with a growth medium. LDH activity was determined in CAR-treated C33A cells according to the manufacturer’s protocol. The LDH activity in treated and untreated cells was determined by evaluating the optical density of the cells at 490 nm using the ELISA reader (Bio-Rad, United States).

#### 2.3.3 Observation of cell morphology

C33A cells plated on 96-well plate were treated with CAR doses (25, 50, 75 and 90 μM) for 24 and 48 h. The CAR-treated and control cells were then observed for morphological alterations within the C33A cervical cancer cells using a fluorescence microscope (Thermo-Scientific, United States of America), and photomicrographs were captured.

#### 2.3.4 Colony formation assay

C33A cells were allowed to attach in each well on a 6-well plate at low counts of approximately 400–500 cells/well and were subsequently used for colony formation assay. Initially, the stated number of C33A cells was exposed to a previously stated concentration of CAR and were left undisturbed under optimum tissue culture conditions for 2 weeks. The colonies were treated and stained using crystal violet stain (0.1%). Aggregates of 40 or more cells were included in the counting as an individual colony.

#### 2.3.5 ROS assay

The intracellular levels of ROS were determined in C33A cervical cancer cells after treatment with various doses of CAR by H_2_DCFDA staining protocol, as described briefly (Ahmad and Ansari, 2021). Briefly, following drug treatment for 12 h, cells were subsequently re-exposed to H_2_DCFDA (25 µM) for 30 min in darkness at 37°C. The fluorescent micrographs were captured under the green fluorescence channel of the FLoid Imaging Station, Thermo-Fischer Scientific, United States.

In addition, ROS generation post-CAR exposure was also quantified through flow cytometric evaluation, as stated previously (Ahmad and Ansari, 2021). 5 × 10^5^ C33A cells were allowed to adhere in each well of a 6-well plate and after that, exposed to state concentrations of CAR as stated above. The cells were then pelleted, resuspended in 25 µM H_2_DCFDA and incubated briefly for 30 min in the dark at RT. Post-incubation, the cells were re-pelleted and re-suspended in PBS (1X). The suspension was eventually analyzed using the FITC channel of the FACSCalibur flow cytometer (BD Biosciences, United States of America).

#### 2.3.6 GSH analysis

Reduced GSH or glutathione levels were quantified using a commercially available GSH kit (BioVision, Mountain View, CA, United States of America) following the supplier’s instructions. Briefly, CAR-treated and untreated C33A cells were exposed to ice-cold GSH buffer (100 µl) for homogenization. The resulting homogenate was placed in a separate test tube containing chilled HClO₄ (10 ml) and vortexed for nearly 1 min. Subsequently, the homogenate was pelleted (13,000 × *g* for 2 min). The supernatant was collected and mixed with KOH in a ratio of 2:1, and after 5 min, the suspension was re-pelleted at the same force (13,000 × *g*), followed by the collection of supernatant for the remaining assay protocol. During the concluding steps, 10 ml of supernatant from different groups was diluted by reconstitution in 80 ml of assay buffer. Finally, the absorbance of fluorescence intensity was read at an excitation/emission ratio of 340/450 nm using a fluorimeter (Thermo-Fischer Scientific, United States of America).

#### 2.3.7 DAPI/PI staining

C33A cells treated with different CAR doses (25, 50, 75, and 90 μM) were collected and fixed in ice-cold methanol for 15 min at −20°C. The cells were stained with DAPI and PI for 30 min at 37°C and analyzed for blue and red merged fluorescence using FLoid Imaging Station, Thermo-Fischer Scientific, United States.

#### 2.3.8 Qualitative staining for apoptosis assessment

CAR-treated and untreated C33A cells at the above-stated concentrations for 24 h were carefully washed using tissue culture grade PBS (1X) and then treated with solution constituted by equal concentrations (100 μg/ml) of Acridine orange (AO) and Ethidium bromide (EtBr) for 30 min. The cells were carefully washed after incubation, visualized, and their red/green merged fluorescence was recorded using FLoid Imaging Station, Thermo-Fischer Scientific, United States.

#### 2.3.9 Assessment of mitochondrial membrane potential (ΔΨm)

C33A cells were exposed to the different concentrations of CAR (25, 50, 75, and 90 μM) for 24 h and then stained with JC-1 dye (200 µM) which gets accumulated within mitochondria following a potential-dependent trend. After that, the cells were pelleted and washed with pre-warmed 1X MMP buffer, and image acquisition was performed by FLoid Imaging Station, Thermo-Fischer Scientific, United States of America. Furthermore, alteration in ΔΨm was quantified using a FACS Calibur flow cytometer (BD Biosciences, United States of America) as per the instructions of the manufacturer JC-1 MMP assay kit. Increased green fluorescence with concomitantly reduced levels of red fluorescence signified dissipated ΔΨm.

#### 2.3.9 Measurement of caspase activities

CAR treatment was given to C33A cells as per above-stated concentration for 24 h in a 96-well plate. The activities of caspase-3, -8 and -9 was assessed using colorimetric kit available commercially and following the instructions supplied by the manufacturer. The observations were interpreted as percentage (%) change in the activities of stated key caspases in comparison with CAR untreated control C33A cells.

#### 2.3.10 Assessment of caspase inhibitors pre-treatment

Cervical cancer cells were initially treated with inhibitors (50 μM; 2 h) specific for caspase-3, -8 and -9, namely Z-DEVD-FMK, Z-IETD-FMK and Z-LEHD-FMK, respectively. Then, C33A cells were treated with the above -stated concentrations of CAR for 24 h. Finally, the viability of cells was calculated using MTT assay as stated in 2.3.1.

#### 2.3.11 PARP estimation

Quantitative assessment of cleaved PARP levels was assessed in C33A cells using a Human PARP ELISA kit as per the manufacturer’s instruction. The absorbance of cleaved PARP was analyzed using a spectrophotometer (Bio-Rad, United States) at 450 nm.

#### 2.3.12 Cytochrome-c release assay

The total concentration of cytochrome-c present within the total protein content of CAR-treated and untreated C33A cells was quantified using an ELISA kit (Thermo-Fischer Scientific, United States) by following the instruction from the manufacturer.

#### 2.3.13 Apoptosis quantification

Apoptosis instigated in C33A cells upon exposure to CAR was quantified through flow cytometry as per the manufacturers’ protocol. Approximately 5 × 10^5^ C33A cells were exposed to the above-stated concentrations of CAR for 24 h. Subsequently, the cells (including the detached ones) were collected and pelleted. Subsequently, the pellets were re-suspended in Annexin V–FITC and PI solution of apoptosis detection kit according to the manufacturer’s recommendations (BD Biosciences, United States). The suspension was then promptly evaluated through FACS Calibur (BD Biosciences, United States), and the interpretation of results was made as a percentage (%) live cells, early apoptotic and late apoptotic or necrotic cells based on Annexin V−, PI−; Annexin V+, PI− and Annexin V+, PI + staining respectively. Moreover, total apoptosis induced in C33A cells was expressed as a percentage of early and late apoptotic C33A cells.

#### 2.3.14 Assessment of cell cycle progression

As previously described, the analysis of cell cycle progression with CAR-treated C33A cells was quantified using a flow cytometer (Ahmad et al., 2022). For the assessment, C33A cells (5 × 10^5^ cells/well) were treated with the above-stated CAR concentrations for 24 h. After that, the cells from each group were trypsinized and fixed using chilled methanol (–20°C for 15 min). Subsequently, the cells were treated with RNase A for 30 min at RT, followed by incubation with PI for 1 h. Eventually, the samples were evaluated through FACSCalibur (BD Biosciences, United States of America.

#### 2.3.15 Real-time PCR (qPCR) analysis

After treatment of 1 × 10^6^ C33A cells/group with the above-stated concentrations of CAR, the total RNA content was isolated using a commercially available RNA isolation kit. The extracted RNA (2 µg) was used to prepare cDNA using the Verso cDNA synthesis kit per the manufacturer’s instructions. qPCR analysis was performed on ABI-7500 real-time PCR (Applied Biosystems) as per the stated instructions of DyNAmoColorFlash SYBR Green qPCR Kit. The sequence of all the primers involved in the investigation was optimized using the NCBI pick tool, as listed in table 1. The normalizations were made using GAPDH as a housekeeping gene and the results were interpreted using the ^2−ΔΔCT^ method.

**TABLE 1:** List of primers used for qPCR.

S. No.	Target gene	Sequence of primers	
		Forward (5′-3′)	Reverse (3′-5′)
1	GAPDH	GAA​ATC​CCA​TCA​CCA​TCT​TCC​AGG	GAG​CCC​CAG​CCT​TCT​CCA​TG
2	Bcl2	GAT​TGT​GGC​CTT​CTT​TGA​G	CAA​ACT​GAG​CAG​AGT​CTT​C
3	Bcl-X_L_	CAG​AGC​TTT​GAA​CAG​GTA​G	GCT​CTC​GGG​TGC​TGT​ATT​G
4	Bax	GCC​CTT​TTG​CTT​CAG​GGT​TT	TCC​AAT​GTC​CAG​CCC​ATG​AT
5	c-myc	AGC​GAC​TCT​GAG​GAG​GAA​CAA​G	GTG​GCA​CCT​CTT​GAG​GAC​CA
6	Cyclin D1	CCGTCCATGCGGAAGATC	GAA​GAC​CTC​CTC​CTC​GCA​CT
7	PTCH1	GGG​TGG​CAC​AGT​CAA​GAA​CAG	TAC​CCC​TTG​AAG​TGC​TCG​TAC​A
8	SMO1	CTA​TTC​ACT​CCC​GCA​CCA​AC	CAG​TCA​GCC​CAC​AGG​TTC​TC
9	GL11	GAA​GTC​ATA​CTC​ACG​CCT​CGA​A	CAG​CCA​GGG​AGC​TTA​CAT​ACA​T
10	Fas	CGG​ACC​CAG​AAT​ACC​AAG​TG	CCAAGTTAGATCTG
11	Fas-L	GGGG	GTGGCCTAT
		ATGTT TCAGCTCTTCC-3	TTG CTT CTCCA
12	CDK4	CCT​GGC​CAG​AAT​CTA​CAG​CTA	ACA​TCT​CGA​GGC​CAG​TCA​TC
13	Bad	CCT​CAG​GCC​TAT​GCA​AAA​AG	AAA​CCC​AAA​ACT​TCC​GAT​GG

#### 2.3.16 *In silico* analysis

To determine the binding effect of the protein and ligands, GLI (PDB ID: 2GLI) and SMO (PDB ID: 4JKV) were docked with Carvacrol (PubChem ID:10,364), Itraconazole (PubChem ID:55,283), and Cyclopamine (PubChem ID: 442,972). All these 3-dimensional structures were retrieved from the PDB database (https://www.rcsb.org/) and PubChem (https://pubchem.ncbi.nlm.nih.gov/). AutoDock Vina four was used for molecular docking. AutoDock Vina is a flexible molecular docking program that has generated nine different docked poses for protein-ligand complexes. The best-docked position was chosen from nine conformations depending on the interacting residues, such as hydrogen bonds with a high binding affinity (kcal/mol). The protein-ligand interaction of docked complexes was presented in two dimensions for interaction analysis of the protein-ligand complex by using LigPlus (Ahmad et al., 2022). The proteins, ligands, and their binding pockets for the protein’s three-dimensional structure were generated using PyMol (Ahmad et al., 2014; Hassan et al., 2014; Baig et al., 2016; Khan et al., 2016; Khan et al., 2021; Khan et al., 2022a; Khan et al., 2022b).

#### 2.3.17 Statistical inferences

The quantitative observations reported are the mean ± SEM of individual experiments performed thrice in triplicate. Statistical significance was calculated after applying one-way ANOVA followed by Dunnett *post hoc* and two-tailed, paired Student’s t-test as per the suitability. *, ** and *** represents *p* < 0.05; *p* < 0.01 and *p* < 0.001 respectively in comparison with untreated control.

## 3 Results

### 3.1 CAR inhibits proliferation and clonogenic potential of C33A cervical cancer cells

**FIGURE 1:**
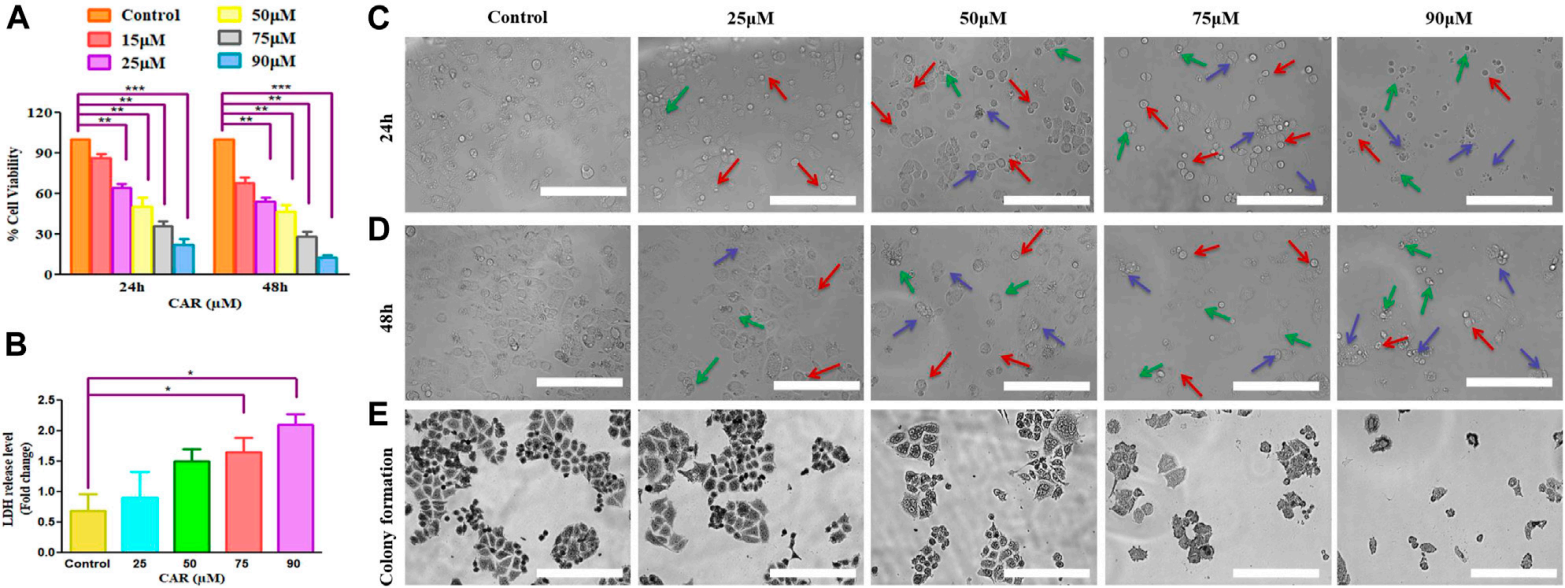
CAR effectively suppressed the growth and colony formation of cervical cancer cells. **(A)** Bar graph represents the percent (%) viability of C33A cells as compared to the vehicle-treated cells after treatment with CAR for 24 and 48 h **(B)** Bar graph represents the percent (%) LDH released as analyzed by LDH assay in CAR-treated C33A cell. **(C,D)**Morphological alterations in C33A cells after treatment with CAR (25–90 µM) for 24 and 48 h as indicated by red, blue and green arrows representing swelling, shrinkage and disintegration of cell organelles. **(E)** CAR treatment reduced the number of colonies in a dose-dependent manner after treatment with various doses of CAR. Photomicrographs were captured at 20X magnification (scale bar = 100 µm). Each value in the bar graph represents the mean ± SEM of three independent experiments. Significant difference among the treatment groups were analyzed by one-way ANOVA followed by Dunnett *post-hoc* test (*p < 0.05, **p < 0.01, ***p < 0.001 represent significant difference compared with control).

We studied the antiproliferative effects of CAR in human cervical cancer cells. C33A cells were cultured with increasing concentrations of CAR (15, 25, 50, 75, and 90 μM) for 24 h. CAR significantly inhibited the viability of C33A cells to 86.47 ± 2.62%, 64.56 ± 2.25%, 50.75 ± 3.28%, 36.39 ± 2.68%, and 22.56 ± 3.02%, respectively, as compared to the control cells, and the population of viable C33A cells declined with increase in CAR concentration (Figure 1A). This effect was more pronounced after treatment of CAR for 48 h and the viability was further reduced to 67.82 ± 3.72%, 54.15 ± 2.50%, 46.81 ± 3.92%, 28.50 ± 2.87%, and 12.56 ± 1.82% (Figure 1A) respectively as compared to the untreated control cells. Subsequently, the release of LDH in CAR-treated C33A cells was quantified. We observed a significant release of LDH to approximately 2.08 folds in C33A cells post-exposure to CAR. Thus, these results of MTT and LDH assays suggested that CAR suppressed the growth and proliferation of cervical cancer cells.

The morphological analysis of CAR-treated C33A cells also showed several morphological alterations compared to the untreated cells. It was observed that the treatment with increasing doses of CAR induced substantial morphological aberrations such as cell shrinkage, rounding of cells, and blebbing of the plasma membrane and disintegration of cell organelles in cervical cancer cells (Figures 1C,D). Thus, CAR exerts antiproliferative effects by inhibiting the proliferation of cervical cancer cells.

Moreover, we performed colony formation assay to study the effect of CAR on cervical cancer C33A cells. Our data showed that C33A cells treated with CAR at indicated concentrations (25–90 µM) for 24 h demonstrated small and few colonies relative to the control cells. Thus, these findings implicated that CAR treatment suppresses cervical cancer cell growth and clonogenic potential (Figure 1E).

### 3.2 Assessment of ROS and GSH levels in CAR-treated C33A cells

**FIGURE 2:**
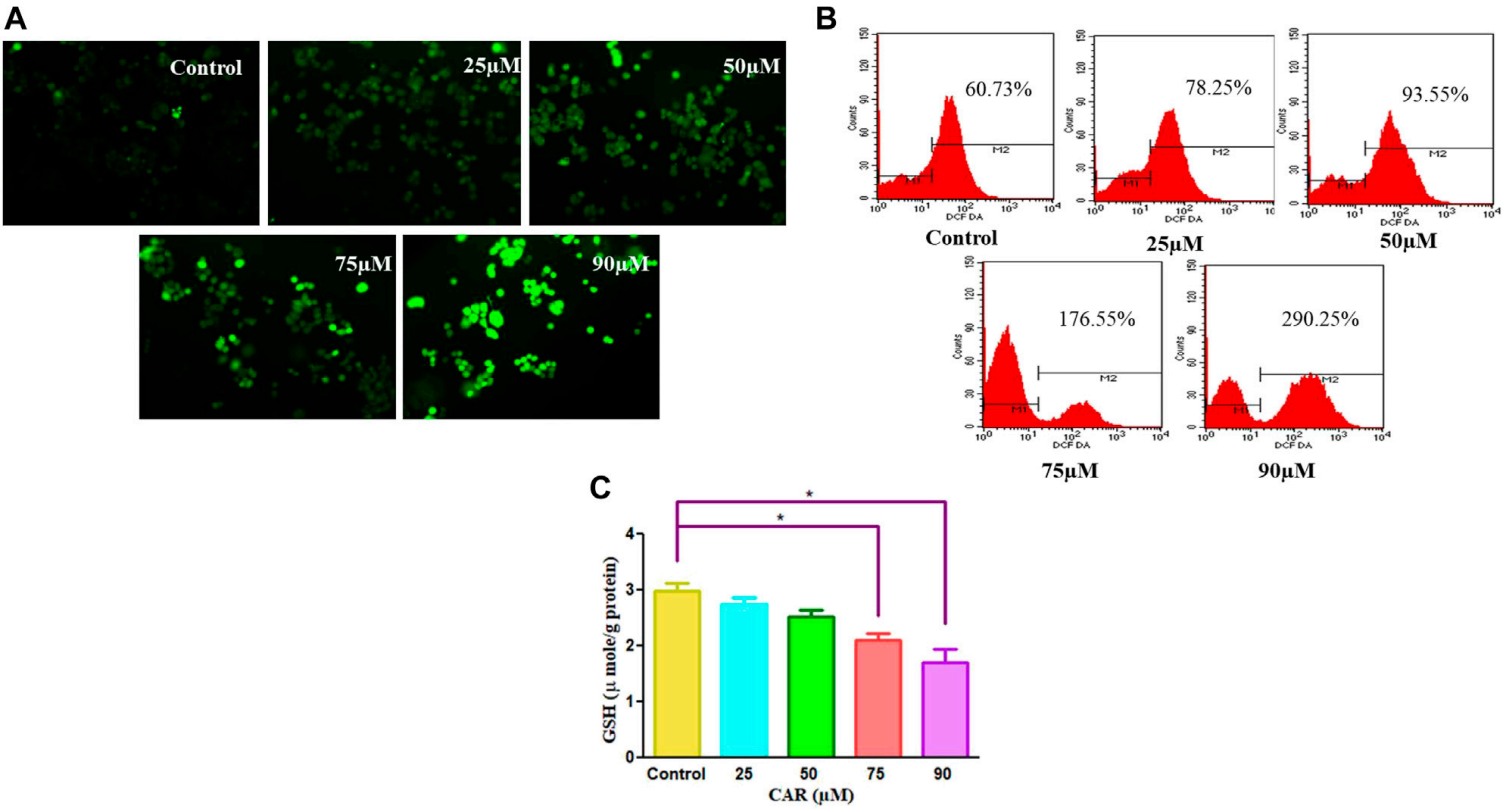
CAR mediates the generation of ROS in cervical cancer cells. **(A)** Fluorescence photomicrographs exhibiting ROS in CAR-treated C33A cells stained with DCFH-DA dye **(B)** Flow cytometric representation of augmented levels of DCFH-DA dye denoting ROS generation **(C)** Percent (%) mean fluorescence intensity (MFI) of DCFHDA-stained after treatment with various concentrations of CAR (25–90 µM). Fluorescent micrographs were captured at ×20 magnification [scale bar = 100 µm]. Each value in the bar graph represents the mean ± SEM of three independent experiments. Significant difference among the treatment groups were analyzed by one-way ANOVA followed by Dunnett *post hoc* test (**p < 0.05 represent significant difference compared with control).

Several physiological and biochemical processes occurring during homeostatic conditions are responsible for ROS production. It is established that ROS play an imperative role in the pathobiology of multiple diseases (Shamas-Din et al., 2013). ROS-inducing property of CAR in C33A cells was assessed qualitatively and quantitatively by fluorescence microscopy and fluorometrically, respectively. It was observed that the amount ROS levels increased by nearly 5-fold in C33A cells, whereas in the untreated cells, the levels of ROS were found to be 60.73% which augmented to 290.25% in treated C33A cells (Figures 2A,B).

GSH is a well-reputed member of the antioxidant family involved in imparting protection against ROS-mediated injury to the cell by inhibiting lipid peroxidation and eliminating hydrogen peroxide (H_2_O_2_). Thus, to find out the effects of CAR on the cellular redox environment, GSH levels were studied. It was noted that CAR treatment reduced the GSH levels in cervical cancer cells (Figure 2C).

### 3.3 CAR promotes apoptosis in C33A cells

**FIGURE 3:**
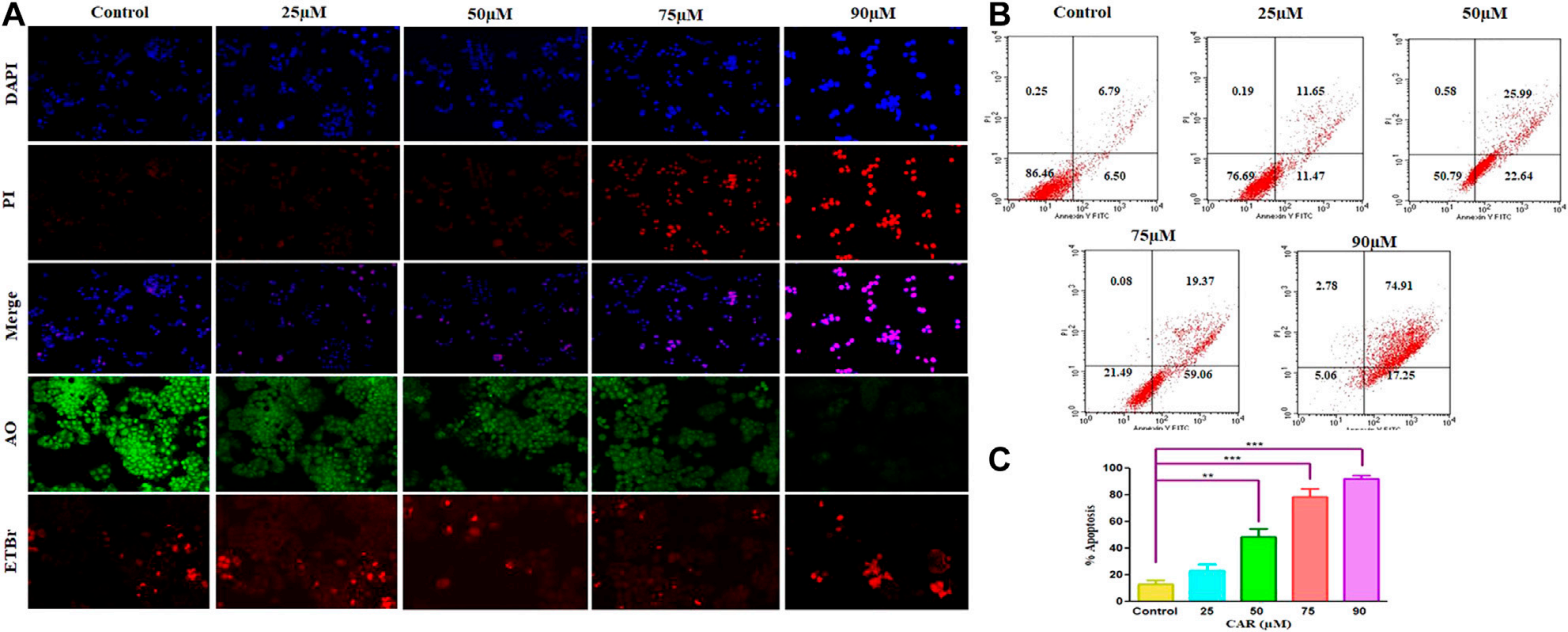
Detection of CAR-mediated apoptosis by fluorescence microscopy in C33A cells. **(A)** C33A cells were treated with various concentrations of CAR for 24 h. Cells were stained with DAPI, PI, AO and EtBr as indicated on the left side of the panels. Fluorescent micrographs were captured at ×20 magnification [scale bar = 100 µm] **(B)** Cells were cultured with various doses of CAR for 24 h and analyzed by flow cytometry using Annexin V and PI dyes. **(C)** Graphical representation of total apoptosis induced in C33A cervical cancer cells during Annexin V-FITC/PI assay. Each value in the bar graph represents the mean ± SEM of three independent experiments. Significant difference among the treatment groups were analyzed by two tailed Paired Student’s t-test and one-way ANOVA followed by Dunnett *post hoc* test (*p < 0.05, **p < 0.01, ***p < 0.001 represent significant difference compared with control).

To delineate the practical implications of CAR treatment as a plausible therapeutic against cervical cancer, cell-based apoptosis was investigated. We primarily studied the morphological alterations under the microscope occurred within the CAR-treated C33A cells. As shown in Figure 3A, treatment with CAR induced condensation, fragmentation and margination of chromatin around the nucleus of C33A cells, which is indicative of programmed cell death evaluated by DAPI/PI staining. An enhanced number of C33A cells were observed exhibiting bright blue and red fluorescence indicating condensed or fragmented nuclei, considered as marked characteristics of apoptosis after the treatment with CAR (Figure 3A).

Furthermore, to investigate the underlying reason for the increase in cell death in cervical carcinoma cells, AO/EB staining was performed in CAR-treated and untreated cervical cancer cells. AO and EB dyes were used to distinguish between live and dead cells based on membrane integrity. AO is reported to infuse within the live DNA and subsequently gives green fluorescence to the cells. Contrastingly, EB uptake occurs only in non-viable cells, where it also infuses in the cellular DNA and gives the nucleus of dead cells characteristic red fluorescence. The fluorescent micrographs, untreated control cells displayed normal morphology of nucleus exhibiting bright green and diffused red fluorescence, whereas CAR-treated cells exhibited bright red fluorescence and diffuse green fluorescence indicating condensed and fragmented nuclei as shown in Figure 3A. Thus, treatment with CAR induced cell death in cervical carcinoma cells.

Moreover, apoptosis was also reaffirmed by performing Annexin V-FITC/PI cells. The total amount of apoptosis in CAR-treated C33A cells was calculated by summation of cells undergoing early and late apoptosis characterized by Annexin V-FITC^+^, PI^−^ and Annexin V-FITC^+^ and PI^+^ respectively. The observations indicated that apoptosis escalated within CAR-treated C33A cells, which was dependent on the concentration and time of exposure to CAR compared with untreated control (Figures 3B,C).

### 3.4 CAR exposure augmented caspase activities in C33A cells

**FIGURE 4:**
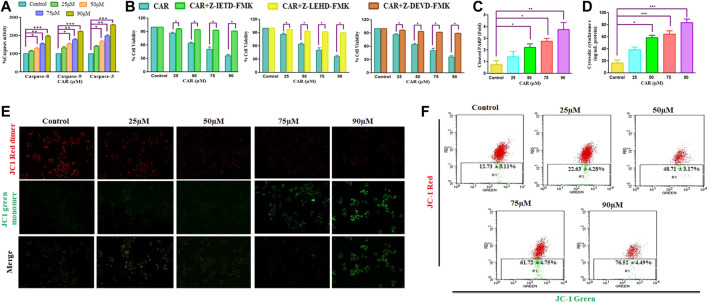
CAR induces apoptosis in C33A cells through caspase activation. **(A)** CAR mediates activation of caspase-8, -9 and -3 **(B)** Percent (%) cell viability of C33AVcells pre-treated with a Z-DEVD-FMK (caspase-3 inhibitor), caspase-8 inhibitor (Z-IETD-FMK) and Z-LEHD-FMK (caspase-9 inhibitor) **(C)** CAR mediates PARP cleavage **(D)** release of cytochrome c in C33A cells **(E)** Fluorescent photomicrographs exhibiting dissipated MMP in JC-1-stained C33A cells after treatment with CAR (25–90 µM) **(F)** Flow cytometric analysis of the ratio of red dimer to green monomer fluorescence. Fluorescent micrographs were captured at ×20 magnification [scale bar = 100 µm]. Each value in the bar graph represents the mean ± SEM of three independent experiments. Significant difference among the treatment groups were analyzed by two tailed Paired Student’s t-test and one-way ANOVA followed by Dunnett *post hoc* test (*p < 0.05, **p < 0.01, ***p < 0.001 represent significant difference compared with control).

Apoptosis, or programmed cell death, is pivotal for maintaining a homeostatic environment within a multicellular organism. Mechanistically apoptotic cell death is primarily classified as intrinsic- and extrinsic apoptosis. The intrinsic apoptosis pathway is also known as the mitochondrial pathway and is characterized by the dissipated potential of mitochondrial membrane leading to apoptosome formation, caspase activation, and activation of downstream effector caspases (caspase-3) (Birben et al., 2012; Zorov et al., 2014). The activities of caspase−3, −8, and −9 was considerably elevated by 41.36 ± 3.30%, 67.46 ± 4.15%, 97.54 ± 4.20%, and 158.67 ± 2.47%; 13.54 ± 4.55%, 29.76 ± 3.93%, 55.05 ± 2.84%, and ± 96.30 ± 3.75% and 32.98 ± 4.12%, 54.06 ± 4.56%, 77.25 ± 4.80%, and 122.51 ± 2.10%, respectively in comparison with CAR untreated control C33A cells (Figure 4A). The data demonstrated that treatment of CAR increased caspase−8, −9 and −3 activities in a dose-proportional manner. Cells were pre-exposed with specific caspase inhibitors to affirm that the activation of caspase is mediated by the treatment of CAR in C33A cells. The result revealed that pretreatment with caspase inhibitors completely abrogated the CAR-induced apoptosis in C33A cells (Figure 4B), which directly indicated that CAR-instigated apoptosis in C33A cells strongly correlates with the activation of key caspases.

Dissipated mitochondrial membrane potential (ΔΨm) is a major event occurring in mitochondria that leads to apoptosis (Shamas-Din et al., 2013). The ΔΨm disruption results in the altered potential of the mitochondrial membrane resulting in imbalanced oxidation-reduction potential within mitochondria. To examine whether CAR could disrupt ΔΨm, membrane-permeant JC-1 dye was used to detect alteration in ΔΨm in CAR-treated C33A cells. JC-1 is highly sensitive to mitochondrial potential, which gives red and green fluorescence for polarized and non-polarized mitochondria. JC-1 exhibits a shift from green to red fluorescence corresponding to approximately 529–590 nm. As a result, the dissipation of ΔΨm is characterized by reduced red: green ratio of JC-1 mediated fluorescence intensity due to the generation of J-aggregates. The merged fluorescent photomicrographs demonstrated that treatment with CAR (25, 50, 75, and 90 µM) for 24 h resulted in mitochondrial depolarization suggested by the decrease in the red to green intensity ratio in C33A cervical carcinoma cells. With the increase in CAR concentration, ΔΨm decreased in cervical cancer C33A cells (Figure 4E). Furthermore, ΔΨm dissipation was also quantitatively measured in CAR-treated cells by flow cytometry (Figure 4F). In C33A cells, 12.73% and 76.52% cell population was found positive for dissipated ΔΨm in CAR untreated control and treated cells respectively, showing a 5.01 fold increase in the depolarized cell population. The results showed that CAR treatment dissipated ΔΨm in C33A cells proportionally with its concentration (Figures 4E,F).

Moreover, treatment of CAR significantly enhanced the level of PARP by 3.94 folds, a well-known marker of apoptosis in a dose-related manner, along with an enhanced level of cytochrome c by four folds in C33A cells, validating the involvement of mitochondria in CAR-induced apoptosis (Figures 4C,D).

### 3.4 Crosstalk between cell survival/death genes in CAR treated C33A cells

**FIGURE 5:**
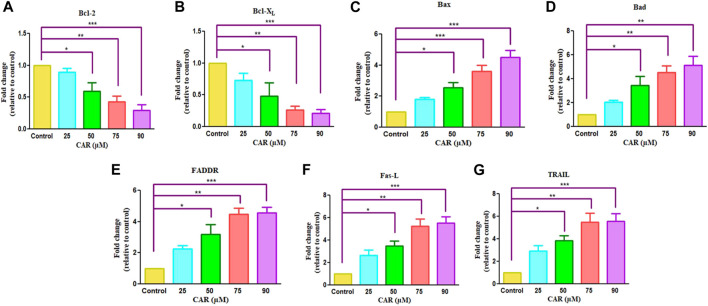
Effect of CAR on the mRNA expression of intrinsic and extrinsic apoptotic signaling molecules in C33A cells. Fold change in the mRNA expression of antiapoptotic proteins and proapoptotic proteins such as **(A)** Bcl-2, **(B)** Bcl-X_L_ **(C)** Bax **(D)** Bad **(E)** FADDR **(F)** Fas-L and **(G)** TRAIL genes were analyzed by real-time PCR using SYBR Green dye. Target gene expression is normalized to GAPDH mRNA expression and the results are expressed as fold change from control. Data reported are mean ± SEM of three separate experiments each of which were performed in triplicates. Significant difference among the treatment groups were analyzed by two tailed Paired Student’s t-test and one-way ANOVA followed by Dunnett *post hoc* test (*p < 0.05, **p < 0.01, ***p < 0.001 represent significant difference compared with control).

The pro- (Bad, Bax) and anti-apoptotic genes (Bcl-X_L_, Bcl-2, Mcl-1) are members of the Bcl-2 protein family that tightly regulates the mitochondrial viability, caspase activation and the release of cytochrome c (Wang et al., 2019). Therefore, we performed real-time PCR to evaluate the mRNA expression of Bcl-2 family proteins. As demonstrated in Figures 5A,B, an increased expression of pro-apoptotic genes with a concomitant decline in anti-apoptotic gene expression after CAR exposure in C33A cells. In treated C33A cells, Bcl-2 and Bcl-X_L_ expression level was decreased (by 0.29 and 0.26 folds), while Bax and Bad expression were elevated (by 4.50 and 5.14 folds) (Figures 5C,D). The exposure of C33A cells with CAR considerably elevated pro-apoptotic gene expression and significantly reduced anti-apoptotic gene expression. These findings revealed that members of the Bcl-2 family played a central role during CAR-mediated apoptotic cell death in C33A cells.

The extrinsic pathway is initiated through transmembrane death receptors through the involvement of FasL and TRAIL. These activate the death receptor with subsequent downstream activation of FADDR and caspase-8, resulting in the onset of apoptosis. After treatment of C33A cells with increased mRNA levels of FasL, TRAIL and FADDR by 5.26, 4.26 and 5.46 folds expression in CAR-treated C33A cervical cancer cells (Figures 5E–G).

### 3.6 Quantification of G0/G1 phase population in CAR-treated C33A cells

**FIGURE 6:**
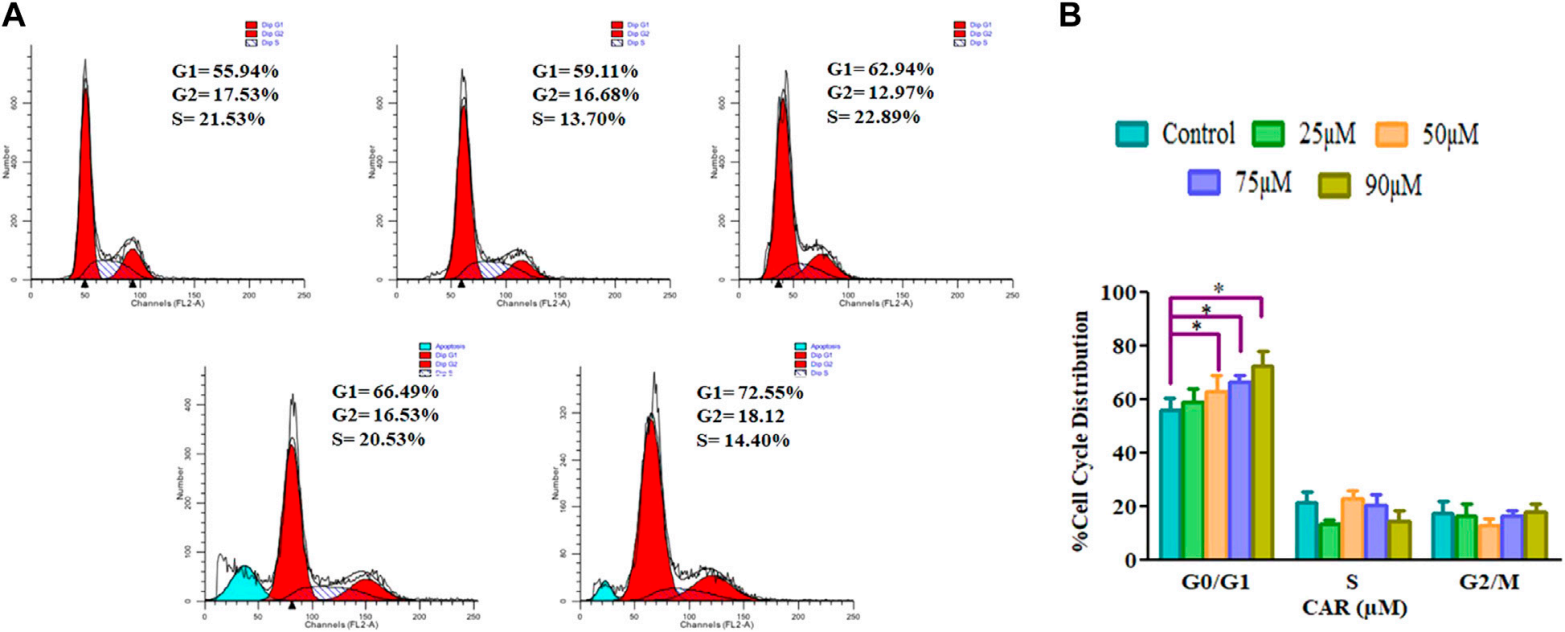
CAR-induced cell cycle arrest in cervical cancer cells **(A)** Cell cycle distribution of propidium iodide-stained C33A cells treated with CAR (25–90 μM) for 24 h observed by flow cytometric analysis. Data shown are representative of three independent experiments. **(B)** Graphical representation of percent cell cycle distribution in C33A cervical carcinoma cells as determined by flow cytometric analysis. Data reported are mean ± S.E.M of three individual experiments performed in triplicate. Significant difference among the treatment groups were analyzed by one-way ANOVA followed by Dunnett post-hoc test (**p < 0.05 represent significant difference compared with control).

**FIGURE 7:**
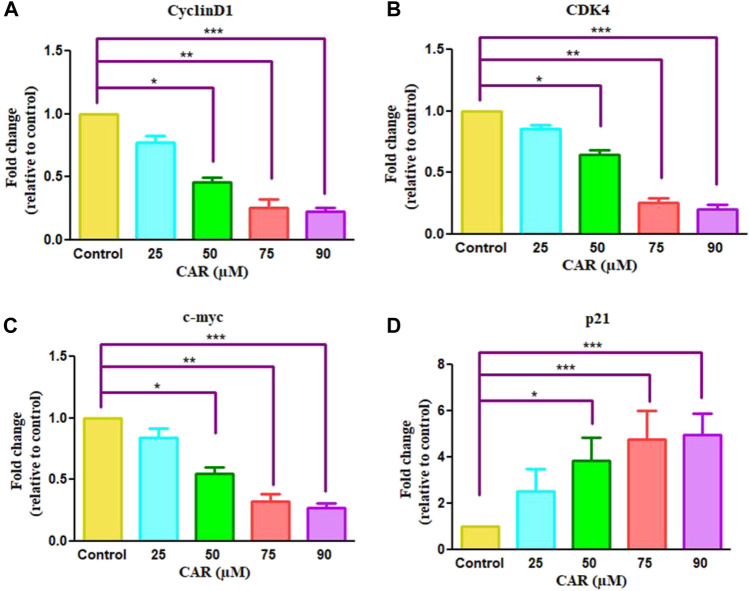
Effect of CAR on the mRNA expression of cell cycle regulatory genes in C33A cells. Fold change in the mRNA expression of antiapoptotic proteins and pro-apoptotic proteins such as **(A)** cyclinD1 **(B)** CDK4 **(C)** c-myc and **(D)** p21genes were analyzed by real-time PCR using SYBR Green dye. Target gene expression is normalized to GAPDH mRNA expression and the results are expressed as fold change from control. Data reported are mean ± SEM of three separate experiments each of which were performed in triplicates. Significant difference among the treatment groups were analyzed by two tailed Paired Student’s t-test and one-way ANOVA followed by Dunnett *post hoc* test (*p < 0.05, **p < 0.01, ***p < 0.001 represent significant difference compared with control).

Cell cycle arrest is a plausibly effective therapeutic target for the clinical management of various cancers (Chen et al., 2002). Exposure of C33A cells to CAR increased the number of C33A cells by 59.11 ± 4.10%, 62.94 ± 5.19%, 66.49 ± 3.27% and 72.55 ± 4.55% in the G0/G1 phase of the cell cycle, respectively comparatively with untreated control C33A cells (55.94 ± 3.79%) (Figures 6A,B). The qRT-PCR analysis also reaffirmed the above results by indicating that CAR exposure decreased cyclin D1, c-myc and cyclin-dependent kinase 4 (CDK4) expression in C33A cells. Furthermore, our data also exhibited that CAR treatment dose-proportionally enhanced the mRNA levels of p21 in C33A cells as compared to untreated groups (Figures 7A–D).

### 3.7 CAR impeded HH/GLI signaling and mediated loss of C33A cell viability

**FIGURE 8:**
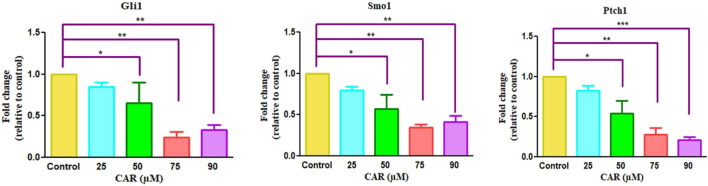
Effect of CAR on mRNA expression of key components of HH signaling pathway in cervical carcinoma cells. The graphs show fold change in mRNA expression of GLI1, SMO1 and PTCH1 relative to control in C33A cells after treatment with CAR (25–90 µM) as measured by qPCR using SYBR Green dye. Target gene expression is normalized to GAPDH mRNA expression and the results are expressed as fold change from control. Data reported are mean ± SEM of three separate experiments each of which were performed in triplicates. Significant difference among the treatment groups were analyzed by two tailed Paired Student’s t-test and one-way ANOVA followed by Dunnett *post hoc* test (*p < 0.05, **p < 0.01, ***p < 0.001 represent significant difference compared with control).

To investigate the involvement of CAR-mediated inhibition of the HH signaling pathway in the underlying molecular mechanism of CAR on C33A cervical cancer cells, we studied the mRNA expression of key components of the HH signaling pathway by qRT-PCR analysis. As the results shown in Fig., CAR treatment (25–90 μM) for 24 h reduced GLI1 and SMO mRNA levels in C33A cells dose-dependently. The reduction in mRNA levels of GLI1 and SMO was found to be 0.84 ± 0.04, 0.65 ± 0.19, and 0.24 ± 0.05 fold; 0.79 ± 0.03, 0.57 ± 0.13, and 0.34 ± 0.03 fold for 25, 50, 75, and 90 μM of CAR treatment respectively in comparison to the control cells (Figures 8A,B). Moreover, CAR treatment also reduced the mRNA expression of PTCH1 by 0.82 ± 0.05, 0.54 ± 0.12, and 0.27 ± 0.06 fold in C33A cervical cancer cells (Figure 8C). Thus, the results suggested that CAR inhibited hyper activated HH signaling in C33A cervical cancer cells.

### 3.4 CAR showed high binding affinity toward GLI and SMO protein

**FIGURE 9:**
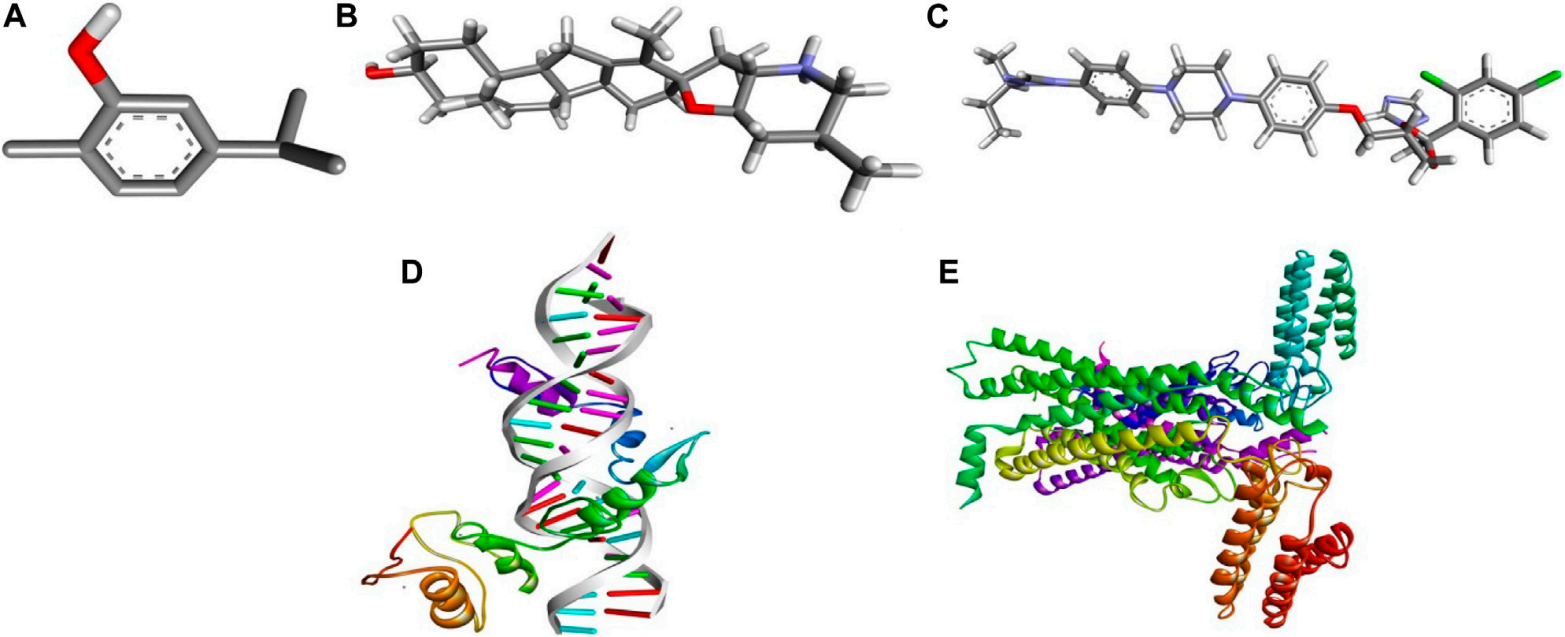
3D chemical structures of **(A)** Carvacrol (CAR) **(B)** Cyclopamine and **(C)** Itraconazole and crystal structures of **(D)** GLI and **(E)** SMO.

Cyclopamine is a steroidal alkaloid with intrinsic teratogenic and anticancer attributes, which results from its ability to impede HH signaling. Cyclopamine inhibits HH pathway activation by binding directly to SMO (Deng et al., 2020). Furthermore, itraconazole is an antifungal drug primarily used as an antimycotic. Recent reports have demonstrated the efficacy of Cyclopamine in impeding HH signaling by inhibiting smoothened receptors (SMO), glioma-associated oncogene homologs (GLI), and subsequent downstream effectors. The 3D structures of ligands of interest, such as carvacrol (CAR), Cyclopamine and itraconazole, are shown in Figures 9A–C. The crystal structures of proteins such as GLI and SMO are depicted in Figures 9D,E. Initially, different types of interaction studies were performed between CAR, Cyclopamine and itraconazole with GLI and SMO. In this study, we have taken Cyclopamine and itraconazole as a reference to compare the interaction of CAR with GLI and SMO. It was observed that CAR showed high binding affinity towards SMO with a binding energy value of -6.9 kcal/mol.

**FIGURE 10:**
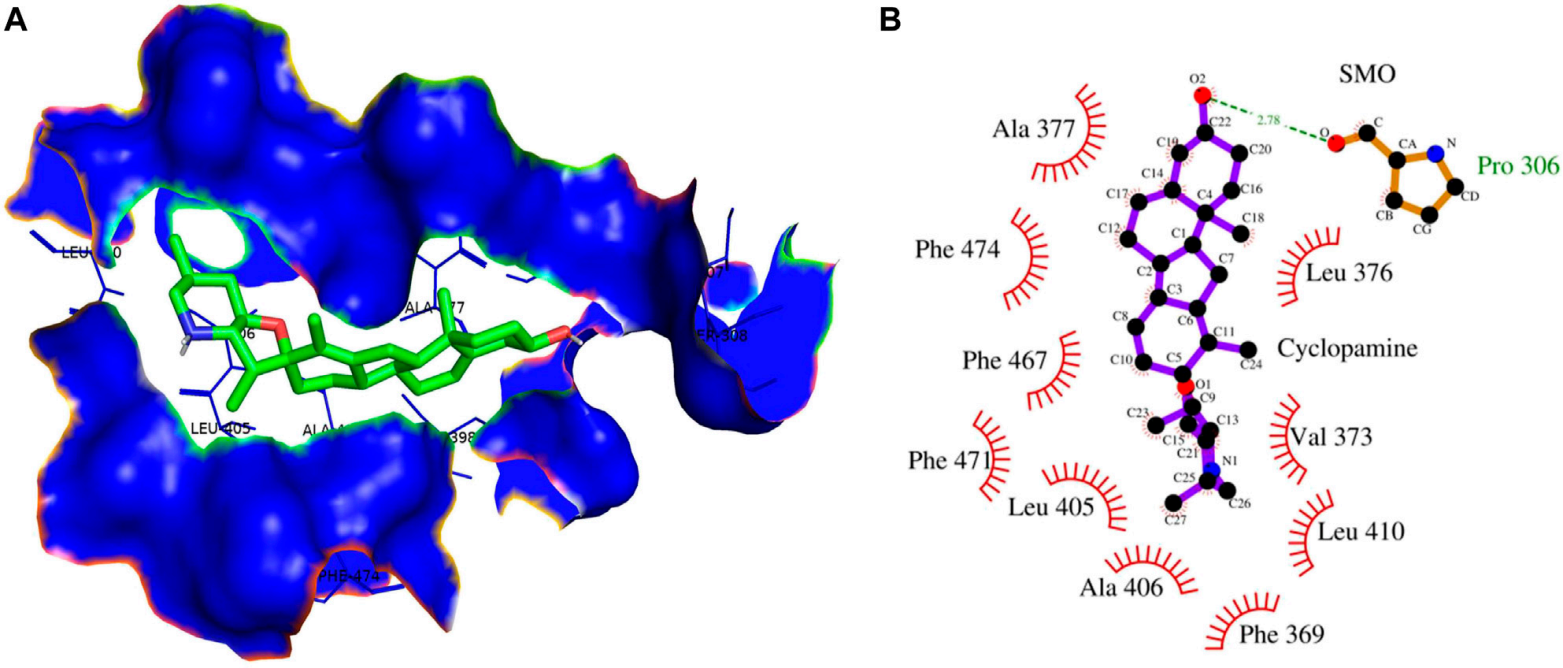
**(A)** 3D Interaction complex of cyclopamine with SMO protein; where blue shows the hydrophobic interactions and green shows the ligand molecule **(B)** 2D Interaction complex of cyclopamine with SMO protein; where red shows the hydrophobic interactions and purple shows the ligand molecule.

**FIGURE 11:**
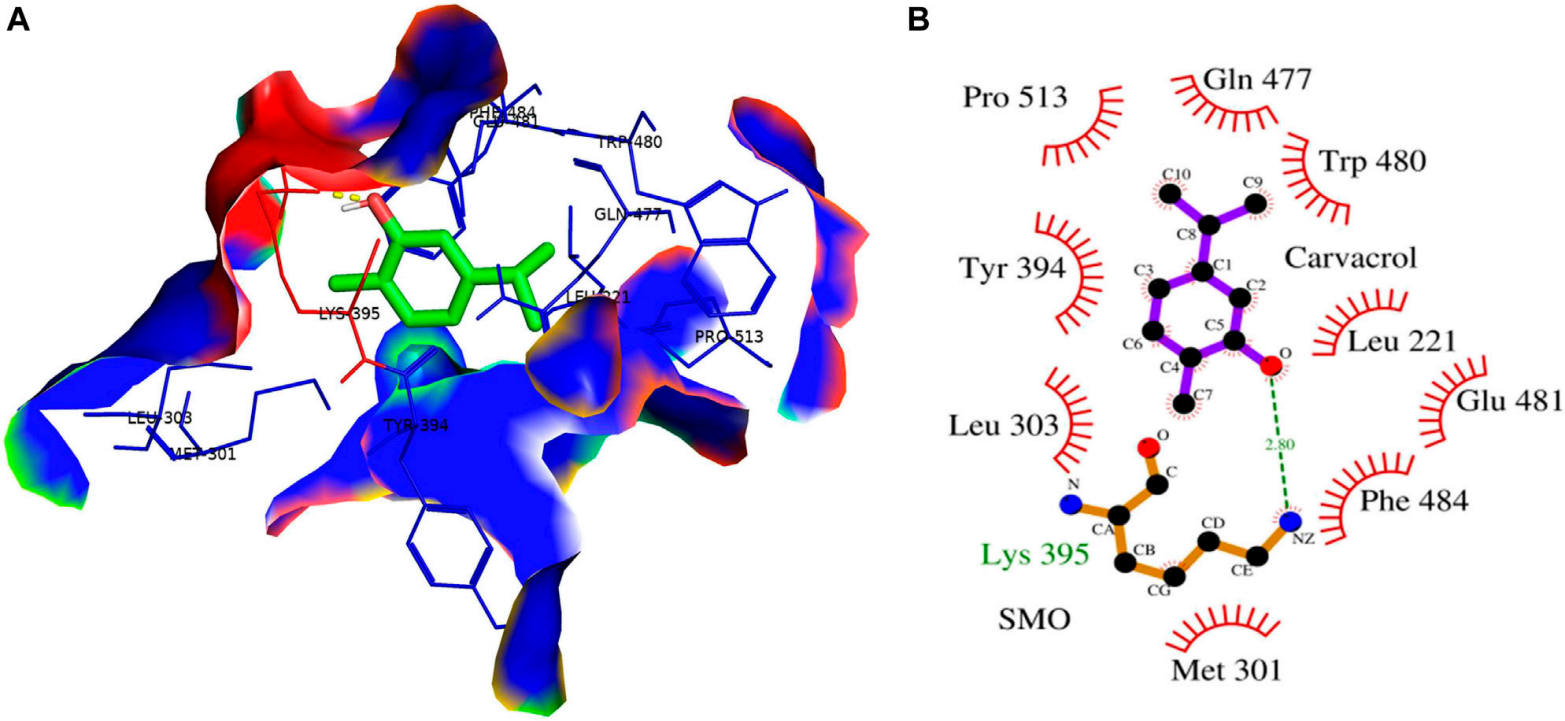
**(A)** 3D Interaction complex of carvacrol (CAR) with SMO protein; where blue shows the hydrophobic interactions and green shows the ligand molecule **(B)** 2D Interaction complex of carvacrol (CAR) with SMO protein; where red shows the hydrophobic interactions and purple shows the ligand molecule.

**FIGURE 12:**
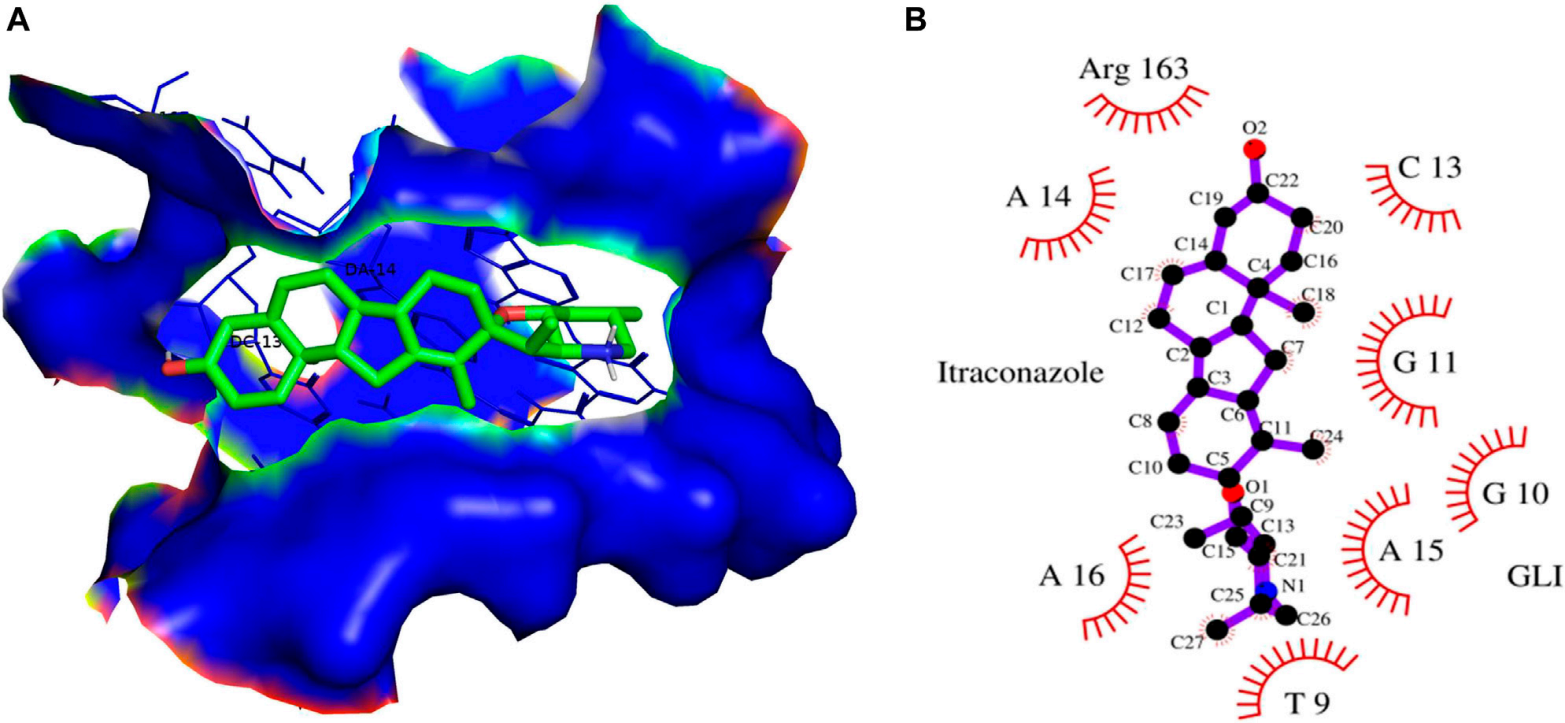
**(A)** 3D Interaction complex of itraconazole with GLI-DNA protein; where blue shows the hydrophobic interactions and green shows the ligand molecule **(B)** 2D Interaction complex of itraconazole with GLI-DNA; where red shows the hydrophobic interactions and purple shows the ligand molecule.

**FIGURE 13:**
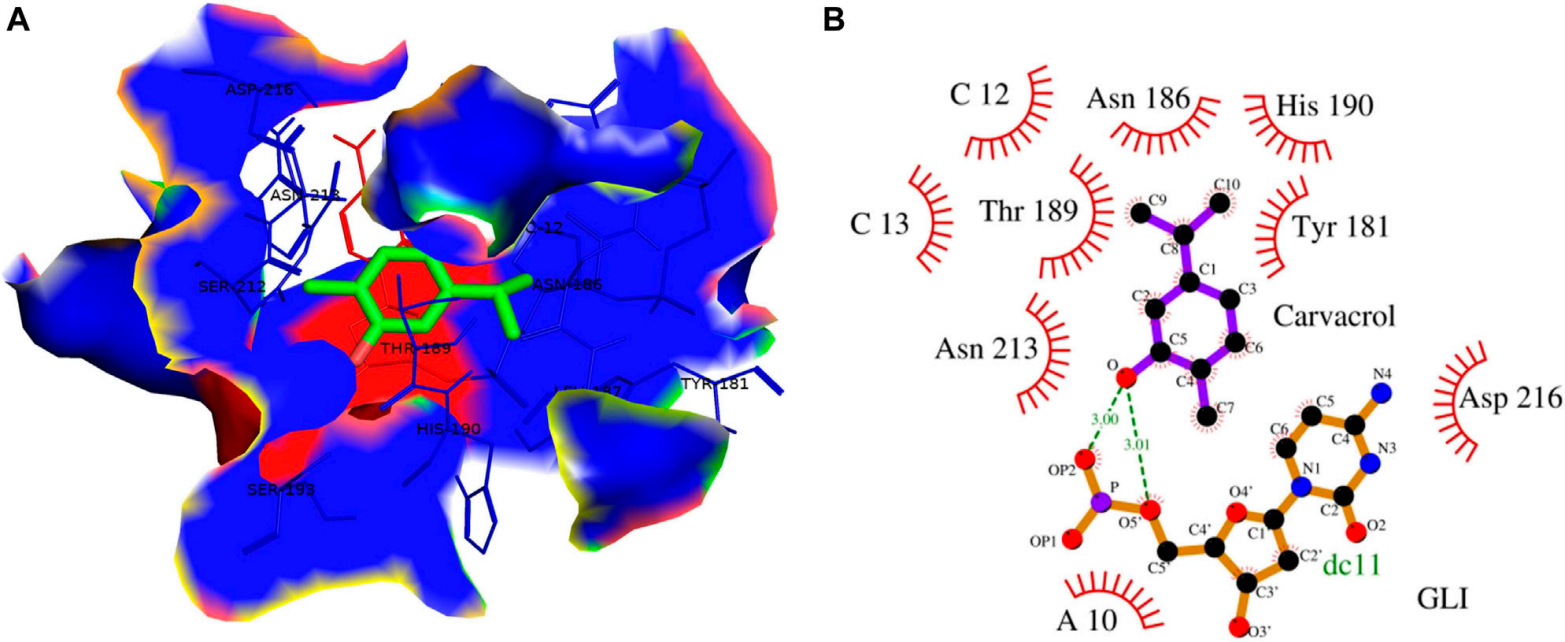
**(A)** 3D Interaction complex of carvacrol with GLI-DNA protein; where blue shows the hydrophobic interactions and green shows the ligand molecule **(B)** 2D Interaction complex of carvacrol with GLI-DNA; where red shows the hydrophobic interactions and purple shows the ligand molecule.

**FIGURE 14:**
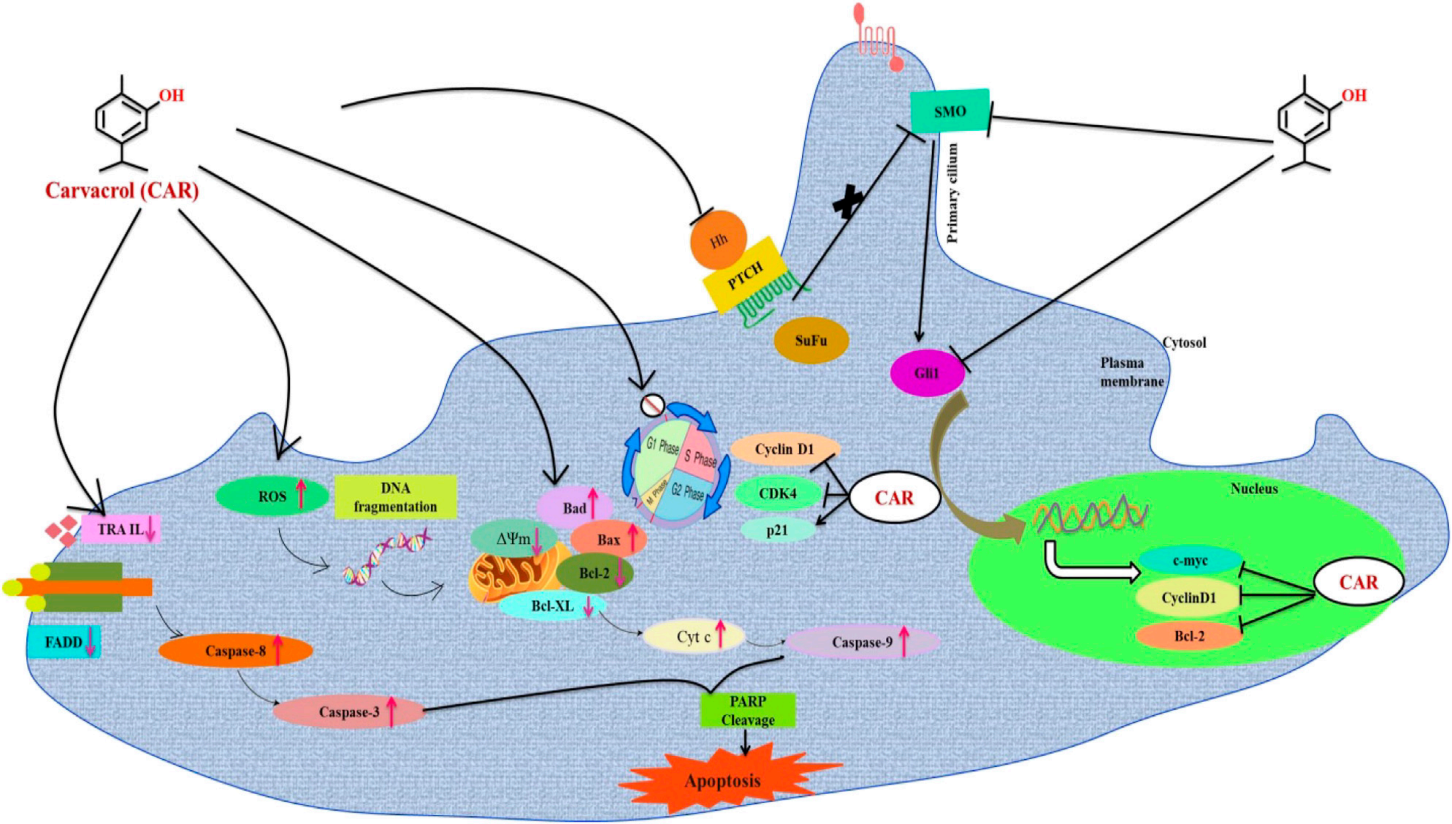
Schematic representation of the mechanism of action of CAR against cervical cancer. CAR downregulates the gene expression of key components of HH pathway, which might be associated with the cell cycle arrest and apoptosis induction in cervical carcinoma cells.

In contrast, Cyclopamine exhibited a binding energy value of -9.3 kcal/mol (Figures 10A,B and Figures 11A,B). Similarly, CAR also showed high binding affinity towards GLI protein with a binding energy of −6.4 kcal/mol. In contrast, itraconazole showed binding energy of −6.4 kcal/mol (Figures 12A,B and Figures 13A,B). However, it is critical to note that the CAR exhibited higher binding energies with SMO and GLI protein, as compared to their standard inhibitors Cyclopamine and itraconazole. Moreover, the amino acid residues of SMO and GLI proteins interacting with Cyclopamine and itraconazole are similar to CAR (Table 2). Furthermore, *in silico* findings concluded that CAR interacted with the GLI-DNA complex to deregulate the HH signaling pathway. However, the exact mechanism has yet to be discovered. Therefore, we tried to corroborate our qPCR results with *in silico* results to establish that CAR downregulated HH signaling pathway and exhibited strong binding affinity towards HH components (SMO and GLI). Furthermore, these results support real-time PCR results and provide a strong rationale for why CAR strongly inhibited the HH signaling in cervical carcinoma cells. The plausible mechanism of action of CAR against cervical cancer is summarized in Figure 14.

**TABLE 2:** Binding energy of carvacrol, cyclopamine and itraconazole with Gli1 and Smo1.

Interacting molecules	Binding energy (Kcal/mole)	Interacting residues
SMO-Cyclopamine	−9.3	Leu^376^, Val^373^, Leu^410^, Phe^369^, Ala^406^, Leu^405^, Gln^477^, Gln^477^, Phe^471^, Phe^467^, Phe^474^, Ala^377^ Pro^306^
SMO-Carvacrol	−6.9	Gln^477^, Trp^480^, Leu^221^, Glu^481^, Phe^484^, Met^301^, Leu^303^, Tyr^394^, Pro^513^, Lys^395^
GLI-Itraconazole	−9.5	Adenine^14,15,16^, guanine^10,11^, thymine^9^, cytosine^13^, Arg^163^
GLI-Carvacrol	−6.4	Cytosine^12,13^, adenine^10^ are the nucleotide, and Tyr^181^, Asn^186^, Thr^189^, His^190^, Asn^213^, Asp^216^

## 4 Discussion

Cervical cancer is the most common gynecological malignancy, which is a reason behind cancer-associated fatalities among women worldwide. Even though numerous screening programs and anticancer therapeutic strategies have improved the prognosis of cervical cancer patients, they still suffer from metastasis and recurrence with a low survival rate. Thus, there is an urge to develop novel drugs or therapeutic interventions to improve patient prognosis.

Earlier reports have outlined the efficacy of CAR in impeding the growth, proliferation and instigation of apoptosis in several cancer cells. In view of these inferences, the investigators of this study focused on elucidating the efficacy of CAR in impeding growth and instigating apoptosis in HPV-negative C33A cells. Rapid proliferation and apoptosis resistance are essential biological attributes of malignant tumors, and most cancer therapies aim to mitigate proliferation and provoke apoptosis on transformed cells. The findings from our practical investigation suggested that CAR was competent in suppressing the proliferation rate of cervical cancer cells. It was observed that CAR exposure significantly reduced the viability of C33A cells as witnessed by MTT and LDH assays, which were well supported by clonogenic assay. Treatment with CAR induces various morphological aberrations within the cervical cancer cells, such as cell shrinkage, blebbing in the cell membrane and detachment of cells from the surface, which collectively indicates apoptosis induction. To our understanding, no study has demonstrated the inhibition of the HH signaling pathway mediating apoptosis induction and cell cycle arrest in cervical cancer cells.

Subsequently, the mechanistic insight of CAR-mediated growth inhibition of cancerous cells was further investigated. From a biochemical perspective, apoptosis is a complex physiological process which is characterized by depolarization as a result of excess ROS generation (Riedl and Salvesen, 2007). Previously, we have reported that CAR could be a plausible therapeutic intervention against HPV-positive cervical cancer cells by targeting ROS-mediated apoptosis and cell cycle arrest (Khosravi-Far and Esposti, 2004). To decipher the functioning of CAR at the molecular level behind its impeding effect on C33A cells, fluorescent microscopy, FACS and qRT-PCR based investigations to ascertain whether the treatment of CAR altered the levels of mitochondrial ROS levels. The results of DAPI/PI and AO/EtBr double staining demonstrated peculiar attributes of apoptosis, including condensation and fragmentation of the nuclei. Further, the increased number of Annexin V-stained cells and cleaved PARP levels in cervical cancer cells indicate that CAR acts as an apoptosis-inducing agent.

Intrinsic apoptosis is instigated by the depolarization of mitochondria, which releases apoptogenic factors such as cytochrome c into the cytosol, eventually mediating the activation of caspase-3 (Jimeno et al., 2008). We found that CAR treatment instigates mitochondria-centered intrinsic apoptosis pathway by activating caspase-3 and -9, dissipation of Δψm with concomitant release of cytochrome c in cervical cancer cells. Additionally, CAR treatment upregulated the mRNA expression of pro-apoptotic proteins and downregulated the expression of anti-apoptotic proteins in cervical cancer cells.

The extrinsic apoptosis is activated through transmembrane death receptors including the targets for FasL and TRAIL. DR4 and DR5 activation primarily culminates in DISC (death-inducing signaling complex) formation by interacting with FADD *via* the death domain. Activation of FADD subsequently results in the recruitment of caspase-8 facilitated by interaction with death effector domains (DEDs), resulting in the activation of caspase-8 (Traverso et al., 2013). In this report, CAR mediates the activation of caspase-8 and increases the mRNA expression level of extrinsic signaling molecules such as FasL, FADDR and TRAIL in C33A cervical cancer cells.

Glutathione (GSH) is pivotal for regulating several processes, such as cell differentiation, cell proliferation, and apoptosis modulation. Intriguingly, alterations within the cellular levels of GSH often lead to the onset and progression of various ailments, including cancer. Elevated levels of GSH are frequently observed in tumors, which subsequently aids in developing chemotherapeutic resistance in neoplastic cells (Circu and Aw, 2012). In the present report, we found a significant depletion of endogenous GSH in C33A cells after the treatment with CAR. Moreover, previous reports have established that GSH depletion is an imperative marker for apoptosis induction in response to various apoptotic stimuli (Thayyullathil et al., 2011). Thus, our data demonstrated that CAR mediates apoptosis in cervical cancer cells, which supports the principle that GSH depletion may favor apoptotic cell death.

Multiple studies have established that targeting drugs/agents responsible for exerting cell cycle arrest could be a plausible therapeutic for treating and managing different cancers, including cervical cancer (Vishnoi et al., 2016). Abrogation of the cell cycle at the G1 phase prevents DNA repair and inhibits entry into S phase. Thus, the G1 checkpoint appeared as a propitious therapeutic target for cancer treatment. The results from this study exhibited that CAR induced a powerful growth-suppressing effect on cervical cancer C33A cells by restricting their progression to G0/G1 phase. Moreover, it is also known that both cyclins and CDKs are pre-requisite for regulating the progression of the cell cycle and their deactivation results in cell cycle arrest. We found inhibitory effects of CAR on cyclin D1 and CDK4 in cervical cancer cells suggesting its interference with cell cycle regulatory proteins. CDK inhibitors, namely p21/WAF1 and p27/KIP1 families of proteins, regulate the CDK activity. Our findings indicate that CAR enhanced the p21 and p27 expression levels in HPV-negative cervical cancer C33A cells. Taken together, the present observations substantiated that CAR was competent in impeding the progression of the cell cycle at G0/G1 phase *via* altering the expression of genes involved in cell cycle regulation in cervical cancer cells. These results further suggested that CAR treatment mediates the inhibition of C33A cells by obstructing the replication of DNA followed by its repair and thereby instigating cell cycle arrest and apoptosis.

The HH/GLI signaling represents an evolutionarily conserved signaling cascade involved in regulating normal development and determination of cell fate. It has been observed that HH signaling contributes to increased chemoresistance, stemness and metastasis. Various components of HH signaling are present in the advanced stages of cervical cancer, indicating that constitutive HH signaling is associated with the development of cervical cancer along with chemoresistance and recurrence (Li et al., 2019). We further studied the CAR-mediated inhibition of HH signaling in C33A cells. The mRNA expression of key proteins such as PTCH1, SMO, and GLI1 was downregulated significantly, indicating that CAR may prevent cervical cancer by modulating hedgehog signaling powerfully.

These findings further intrigued us to investigate the mechanism of CAR-mediated cytotoxic effects in comparison to cyclopamine and itraconazole. The interactions between CAR, cyclopamine and itraconazole with SMO and GLI proteins were studied by *in silico* techniques. It was found that the binding energies (BE) of cyclopamine and SMO protein was -9.3 kcal/mol, which is nearby and comparable to BE of CAR and SMO (-6.9 kcal/mol). Similarly, the BE of itraconazole and GLI protein was -9.5 kcal/mol, whereas CAR and GLI were -6.3 kcal/mol and were also observed near each other. Furthermore, our *in silico* studies have substantiated that the amino acid residues involved in the interaction of CAR with SMO and GLI were considerably similar as compared to the reference drugs (cyclopamine and itraconazole). Thus, our *in silico* findings corroborated RT-PCR results and concluded that CAR could inhibit the HH signaling cascade in cervical cancer cells. After an exhaustive literature review, the authors of the present manuscript are confident that this is the report which demonstrated the CAR-mediated apoptosis *via* inhibition of HH signaling cascade in the C33A cell line. Nevertheless, a more exhaustive study using appropriate *in vivo* models is further warranted to establish the pre-clinical efficacy of CAR against cervical cancer.

## 5 Conclusion

Collectively our findings conclude that CAR principally exerted antiproliferative and apoptotic effects in C33A (HPV-) cervical cancer C33A cells *in vitro* by inducing the production of ROS inside mitochondria of C33A cells, which provoked multiple cellular events leading to mitochondrion-centered intrinsic apoptosis. Furthermore, CAR also modulates the key signaling components of extrinsic or death receptor pathways. Thus, CAR could suppress cell proliferation and induce apoptosis in cervical carcinoma cells by inhibiting HH signaling pathway. The investigators believe that this is a novel report elucidating the best of our understanding. This is the first report demonstrating the antiproliferative and apoptotic potential of CAR against human cervical carcinoma C33A cells *via* targeting the HH signaling pathway. Nevertheless, our present report is still restricted to the *in vitro* evidence; yet due to the significant anticancer potential of CAR, we propose it as a suitable drug for cervical cancer therapy after sufficient pre-clinical and clinical trials.

## Data Availability

The raw data supporting the conclusions of this article will be made available by the corresponding authors after due discussion.
